# Second Law of Thermodynamics and Strain Gradient Theories of Elasticity

**DOI:** 10.3390/e28050487

**Published:** 2026-04-24

**Authors:** Claudio Giorgi, Angelo Morro

**Affiliations:** 1 Dipartimento di Ingegneria Civile Ambiente Territorio Architettura e Matematica, Università di Brescia, Via Diogene Valotti 9, 25133 Brescia, Italy; 2 Dipartimento di Informatica, Bioingegneria, Robotica e Ingegneria dei Sistemi, Università di Genova, Via All’Opera Pia 13, 16145 Genova, Italy; angelo.morro@unige.it

**Keywords:** second law of thermodynamics, Clausius–Duhem inequality, non-local constitutive equations, higher-order elasticity theories, second strain gradient elasticity, 74A15, 74A20, 80A17

## Abstract

The paper addresses the second law of thermodynamics through the Clausius–Duhem inequality in its general form, with entropy flux and entropy production given by suitable constitutive functions. For definiteness the paper investigates possible models of elastic solids where, to account for non-local properties, the stress depends on strain gradients up to second order. While previous approaches are developed through variational formulations or by applying the virtual power method, here it is shown that no change in the energy balance or the form of kinetic energy is necessary; it is sufficient that the entropy flux be given by a suitable constitutive function. The paper also emphasizes that non-local constitutive properties arise from the Clausius–Duhem thermodynamic inequality, while variational formulations and the virtual power method are in fact limited to the purely mechanical context, as they involve only the equation of motion.

## 1. Introduction

The balance of entropy for continuous bodies has long been expressed by the corresponding continuous formulation of the principle of increase of entropy. If η is the specific entropy density, ρ the mass density, q the heat flux, *r* the heat supply and θ the absolute temperature, then in any process, the time derivative $\dot{\eta }$ is subject to

(1) $$\rho \gamma =\rho \dot{\eta }+\nabla \cdot \frac{q}{\theta }-\frac{\rho r}{\theta }\geq 0.$$

Equation (1) is usually referred to as the CD (Clausius–Duhem) inequality. By definition the left-hand side of (1) is called the rate of entropy production, in that ργ gives the excess of the rate $\rho \dot{\eta }$ relative to the entropy flux q/θ and the entropy supply ρr/θ. The assumption that the resulting inequality must hold for every admissible thermodynamic process is due to Coleman and Noll [1]. Consequently, the CD inequality becomes conceptually a criterion for the selection of physically admissible models of continua.

Later on, Müller [2] observed that, for greater generality, the CD inequality should be expressed with an entropy flux j not necessarily equal to q/θ. Then, Equation (1) should be replaced with

(2) $$\rho \gamma =\rho \dot{\eta }+\nabla \cdot j-\frac{\rho r}{\theta }\geq 0.$$

The definition of ργ, in (1) and (2), is usually viewed as being identically equal to the right-hand side. In our mind, it is a further natural generalization of the CD inequality to view ργ as being equal to the right-hand side but not identically equal. In other words, γ might be given by a proper (constitutive) function not necessarily defined by the CD inequality, so that (1) and (2) are viewed as equations, not as identities (see Section 2). In connection with (1), this view has already proved profitable in the modelling of hysteretic materials (see [3] and [4] Chapters 13–15). A further application of this view on γ is given in [5]. In our mind, whenever involved models of materials are considered, which account, e.g., for non-locality in space-time or hysteresis, the CD inequality is required to be in the general form (2) with j and γ as given by appropriate constitutive functions.

Heterogeneous systems and mere phenomena at the micro- and nano-level are modelled by assigning a dependence on gradients of suitable order [6,7].

Practical applications indicate interest in the modelling of elastic solids with a dependence of the stress on strain gradients of various orders [8,9,10,11]. This topic has been investigated in several ways, mainly through variational formulations [12,13] or modifying the balance of energy through the method of virtual power [14].

It is the purpose of this paper to investigate the thermodynamic consistency of strain-gradient models through the corresponding validity of the CD inequality (2), via appropriate entropy flux j and entropy production γ. This purpose is pursued without appealing to the method of virtual power, by assuming the stress power with hyper-stresses, or modifying the definition of kinetic energy within a variational approach.

In this paper we consider possible models of elastic materials with a dependence of the stress on strain gradients. This topic has been investigated in several ways in the literature, mainly through variational formulations or by modifying the balance of energy through the method of virtual power. Our approach is instead based on the CD inequality and is completely original.

It is worth mentioning that different approaches in the literature develop strain-gradient models by following Liu’s procedure [15,16] possibly involving internal variables [17] or hyper-stresses [18]. Some comments are given in Section 7.

## 2. Notation and Balance Equations

Let Ω be the time-dependent region occupied by the body. The position of a point in Ω is denoted by the vector x, relative to a chosen origin *O*, as a function of the time *t* while the position in a reference configuration R is denoted by X. So x=x(X,t), as t∈R, describes the motion of X. For definiteness, the components of the vectors are considered relative to a right-handed orthonormal triplet $e_{1},e_{2},e_{3}$. We denote by ∇ and ∇R the gradient operator with respect to x and X. The gradient ∇Rx is called the deformation gradient and denoted by F; in components $F_{iK}=\partial_{X_{K}}x_{i}$. The difference u=x−X is called the displacement and then H=∇Ru, viz. $H_{iK}=\partial_{X_{K}}u_{i}$ is the displacement gradient and F=1+H, with 1 being the identity tensor. The tensor $E=\frac{1}{2}\left(F^{T}F-1\right)$ is the symmetric Green–Lagrange strain. If, for any entry $H_{iK}$, it is $|H_{iK}|\ll 1$, then F≃1 and

∇Ru=∇uF≃∇u.

Hence, if $|H_{iK}|\ll 1$ then the strain E may be approximated by

$$E=\frac{1}{2}\left(H^{T}+H+H^{T}H\right)≃\mathrm{sym}\nabla u=:\epsilon ,$$

with sym denoting the symmetric part, $\mathrm{sym}\nabla u=\frac{1}{2}\left[\nabla u+{\left(\nabla u\right)}^{T}\right]$. The tensor ϵ is called infinitesimal strain.

To save writing, we denote partial spatial derivatives by a comma followed by a suffix; e.g., $f_{,j}$ stands for $\partial_{x_{j}}f$, j=1,2,3.

A superposed dot denotes the material (or Lagrangian) time derivative. For any function φ(x,t) it is

$$\dot{\varphi }=\partial_{t}\varphi +\left(v\cdot \nabla \right)\varphi ,$$

where $v=\dot{x}$ is the velocity. The tensor L denotes the velocity gradient, $L_{ij}=v_{i,j}$, and D=symL.

Let ρ be the mass density. The conservation of mass results in the continuity equation

(3) $$\dot{\rho }+\rho \nabla \cdot v=0\;\mathrm{or}\;\partial_{t}\rho +\nabla \cdot \left(\rho v\right)=0.$$

Let T be the Cauchy stress tensor and b the body force per unit mass. The balance of linear momentum results in the equation of motion

(4) $$\rho \dot{v}=\nabla \cdot T+\rho b,$$

where

$${\left(\nabla \cdot T\right)}_{i}=\partial_{x_{j}}T_{ij}.$$

We assume the body is non-polar. The balance of angular momentum then implies that

(5) T∈Sym,

where Sym is the set of symmetric tensors.

The balance of energy is considered by letting ε be the non-kinetic energy density per unit mass; *r* is the heat supply and q the heat flux. Hence, the balance of energy density $\rho (\frac{1}{2}v^{2}+\varepsilon )$ results in the equation

$$v\cdot \left(\rho \dot{v}-\nabla \cdot T-\rho b\right)+\rho \dot{\varepsilon }-T\cdot D-\rho r+\nabla \cdot q=0.$$

Using the equation of motion (4) we conclude that the balance of ε reads

(6) $$\rho \dot{\varepsilon }=T\cdot D+\rho r-\nabla \cdot q.$$

Let θ be the absolute temperature and η the entropy density per unit mass. The balance of entropy is assumed by asserting that the time derivative $\rho \dot{\eta }$, deprived of the entropy supply ρr/θ and the contribution of the entropy flux j, is non-negative. Formally we require that

(7) $$\rho \dot{\eta }-\frac{\rho r}{\theta }+\nabla \cdot j=\rho \gamma ,$$

where j is the entropy flux vector to be established and γ≥0 is the entropy production density per unit mass. For technical convenience let

$$j=\frac{q}{\theta }+k$$

where q/θ is the classical entropy flux, arising from the thermodynamic of systems, and k is the extra-entropy flux to be determined depending on the material. Replacing the expression of j we have

$$\rho \dot{\eta }+\frac{1}{\theta }\left(\nabla \cdot q-\rho r\right)-\frac{1}{\theta^{2}}q\cdot \nabla \theta +\nabla \cdot k=\rho \gamma \geq 0.$$

A thermodynamic process is the set of time-dependent fields

ρ,x,T,θ,ε,b,r,q,η,γ

on Ω×R, that satisfy the balance Equations (3)–(6). As the second law of thermodynamics we assume that *for any physically admissible thermodynamic process the Equation (7) holds with γ non-negative*. In view of (6) we replace ρr−∇·q and consider the Helmholtz free energy ψ=ε−θη to obtain

(8) $$-\rho \left(\dot{\psi }+\eta \dot{\theta }\right)+T\cdot D-\frac{1}{\theta }q\cdot \nabla \theta +\theta \nabla \cdot k=\rho \theta \gamma .$$

Throughout, it is understood that γ≥0 and, consistent with the literature, Equation (8) is referred to as the CD (Clausius–Duhem) inequality. Since the number of functions is larger than the number of balance equations then a thermodynamic process is completed by a set of constitutive equations. Hence the CD inequality results in a selection procedure of physically admissible constitutive equations.

The validity for any thermodynamic process implies that the inequality is a constraint on the constitutive equations. This general feature is investigated in [15,19] in connection with the equivalence between thermodynamic equilibrium and reversible processes.

The exploitation of the second law has been widely developed through the so-called Coleman–Noll procedure where the fields b and *r* are taken to be arbitrary time-dependent fields. Instead, in the Liu approach [20] both b and *r* are zero and then all balance equations are viewed as constraints. In both procedures the entropy production γ is a constitutive function defined by (7). In the following sections of this paper γ turns out to be given by (7) as it happens usually in the literature. Yet, mainly in view of hysteretic models, we follow a generalization of the Clausius–Duhem inequality, by letting γ be given by a proper non-negative constitutive function, just as the entropy flux j is allowed to be given by a constitutive function and not necessarily by q/θ.

To describe micro- and nano-scale objects, a wide range of literature has been developed with the dependence of the stress tensor on gradients of the strain (see, e.g., Refs. [6,7,8] and references therein). In our view, the dependence on strain gradients should not imply an arbitrary change of balance Equations (3)–(6). This is a crucial feature and we now examine its conceptual aspects in detail.

## 3. Higher-Order Elasticity Through Hyper-Stresses

Based on the sometimes tacit assumption that $|H_{iK}|\ll 1$ the strain gradient elasticity involves as variables the tensors ϵ,∇∇u,∇∇∇u or, in suffix notation,

$$\epsilon_{ij}=\frac{1}{2}\left(u_{i,j}+u_{j,i}\right),\;u_{i,jk}\;u_{i,jhk}.$$

The dependence of constitutive relations on strain gradients was motivated by Mindlin [21] to describe capillarity also in connection with Korteweg’s fluid.

The starting point is the definition of third-gradient material as a solid with a free energy ψ that depends on ϵ,∇∇u, and ∇∇∇u. Hence, apart from other dependencies such as, e.g., the temperature, we have

$$\rho \dot{\psi }=\rho \partial_{\epsilon_{ij}}\psi {\left(\epsilon_{ij}\right)}^{˙}+\rho \partial_{u_{i,jk}}\psi {\left(u_{i,jk}\right)}^{˙}+\rho \partial_{u_{i,jhk}}\psi {\left(u_{i,jhk}\right)}^{˙}.$$

Upon the definitions

$$\sigma_{ij}=\rho \partial_{\epsilon_{ij}}\psi \in \mathrm{Sym},\;T_{ijk}=\rho \partial_{u_{i,jk}}\psi ,\;S_{ijhk}=\rho \partial_{u_{i,jhk}}\psi ,$$

and following the general statement on the virtual power [14], the power of internal forces in a region V is taken as (see, e.g., [7])

(9) $$P=\int_{V}\left[\sigma_{ij}{\left(\epsilon_{ij}\right)}^{˙}+T_{ijk}{\left(u_{i,jk}\right)}^{˙}+S_{ijhk}{\left(u_{i,jhk}\right)}^{˙}\right]dv,$$

with the tensors T and S being referred to as hyper-stresses.

We notice that (see, e.g., [4] Section 1.5.1)

(10) $${\left(u_{i,j}\right)}^{˙}={\dot{u}}_{i,j}-L_{pj}u_{i,p}$$

and likewise for ${\left(u_{i,jk}\right)}^{˙},{\left(u_{i,jhk}\right)}^{˙}$. To find a connection with the literature [7], here we follow a frequently adopted claim, namely

$${\left(u_{i,j}\right)}^{˙}\approx {\dot{u}}_{i,j},\;{\left(u_{i,jk}\right)}^{˙}\approx {\dot{u}}_{i,jk},\;{\left(u_{i,jhk}\right)}^{˙}\approx {\dot{u}}_{i,jhk},$$

where ≈ denotes that, according to (10), $L_{pj}\partial_{x_{p}}$ terms are neglected. Using this claim we compute

$$\sigma_{ij}{\left(\epsilon_{ij}\right)}^{˙}=\sigma_{ij}{\left(u_{i,j}\right)}^{˙}\approx \sigma_{ij}{\dot{u}}_{i,j}={\left(\sigma_{ij}{\dot{u}}_{i}\right)}_{,j}-\sigma_{ij,j}{\dot{u}}_{i},$$

Likewise,

$$\begin{array}{c} T_{ijk}{\left(u_{i,jk}\right)}^{˙}\approx T_{ijk}{\dot{u}}_{i,jk}={\left[T_{ijk}{\dot{u}}_{i,j}\right]}_{,k}-T_{ijk,k}{\dot{u}}_{i,j}\\ ={\left[T_{ijk}{\dot{u}}_{i,j}\right]}_{,k}-{\left[T_{ijk,k}{\dot{u}}_{i}\right]}_{,j}+T_{ijk,jk}{\dot{u}}_{i}], \end{array}$$

$$\begin{array}{c} S_{ijhk}{\left(u_{i,jhk}\right)}^{˙}\approx S_{ijhk}{\dot{u}}_{i,jhk}={\left[S_{ijhk}{\dot{u}}_{i,jh}\right]}_{,k}-S_{ijhk,k}{\dot{u}}_{i,jh}\\ ={\left[S_{ijhk}{\dot{u}}_{i,jh}\right]}_{,k}-S_{ijhk,hk}{\dot{u}}_{i,j}={\left[S_{ijhk}{\dot{u}}_{i,jh}\right]}_{,k}-{\left[S_{ijhk,k}{\dot{u}}_{i,j}\right]}_{,h}+S_{ijhk,hk}{\dot{u}}_{i,j}\\ ={\left[S_{ijhk}{\dot{u}}_{i,jh}\right]}_{,k}-{\left[S_{ijhk,k}{\dot{u}}_{i,j}\right]}_{,h}+{\left[S_{ijhk,hk}{\dot{u}}_{i}\right]}_{,j}-S_{ijhk,jhk}{\dot{u}}_{i}. \end{array}$$

Based on these identities we can represent the claimed power P as the sum of body terms and boundary terms in the form

$$\begin{array}{c} P=-\int_{V}\left[\sigma_{ij,j}-T_{ijk,jk}+S_{ijhk,jhk}\right]{\dot{u}}_{i}dv+\int_{\partial V}{\dot{u}}_{i}\left[\sigma_{ik}-T_{ijk,j}+S_{ijhk,jh}\right]n_{k}\;da\\ +\int_{\partial V}\left\{{\dot{u}}_{i,j}\left[T_{ijk}-S_{ijhk,h}\right]+{\dot{u}}_{i,jh}S_{ijhk}\right\}n_{k}da \end{array}$$

Accordingly, the assumption (9) about the power associated with the stress tensors σ,T,S results in the effective body force

∇·σ−∇·(∇·T)+∇·(∇·(∇·S)).

As usual in mechanics, the divergence of a *n*-order tensor is a (n−1)-order tensor calculated by summing the partial derivatives over the last index.

Some comments are in order about the introduction of hyperstresses in the continuum modelling. Mainly because of variational approaches involving an energy function with a dependence on the first strain gradient [22,23], the conclusion follows that the effective stress $\hat{\sigma }$ is in the form [8,13]

$$\hat{\sigma }=\sigma -\nabla \cdot \tau ,\;\sigma =\partial_{\epsilon }W,\;\tau =\partial_{\nabla \epsilon }W.$$

More generally [6,12,21], using the second strain gradient in the form

$$\epsilon_{1}=\epsilon ,\;\epsilon_{2}=\nabla \nabla u,\;\epsilon_{3}=\nabla \nabla \nabla u,$$

or

$$\epsilon_{1}=\epsilon ,\;\epsilon_{2}=\nabla \epsilon ,\;\epsilon_{3}=\nabla \nabla \epsilon ,$$

the effective stress $\hat{\sigma }$ is given by

$$\hat{\sigma }=\partial_{\epsilon_{1}}W-\nabla \cdot \partial_{\epsilon_{2}}W+\nabla \cdot \left(\nabla \cdot \partial_{\epsilon_{3}}W\right).$$

The method of virtual power by Germain [14] has given a formal basis to the representation of the power of internal forces as linear forms with respect to strain gradients, as in (9) for elastic materials, or velocity gradients for applications to fluids (see, e.g., [24,25,26,27]).

In the next section we prove that a thermodynamic procedure holds without any introduction of hyper-stresses. We show that strain gradient elasticity may be modelled in a deeply different thermodynamic scheme by involving a single stress tensor and thus avoiding the split of the mechanical power in the form (9).

## 4. Higher-Order Strain Gradient Elasticity via a Single Stress Tensor

Based on Cauchy’s theorem about the existence of a stress tensor T, for non-polar bodies it is $T=T^{T}$ and the stress power is taken as

$$P=\int_{\partial \Omega }v\cdot Tn\;da.$$

It then follows that T·D is the body power density acting on the internal energy ρε. Two schemes are now established that are based on the use of the stress power T·D. One is Eulerian-like with the Cauchy stress T though with a strain derivative in place of D. The other one is Lagrangian with the stress state described by the second Piola stress

$$T_{RR}=JF^{-1}TF^{-T}.$$

### 4.1. Cauchy Stress Through Strain Gradients

Consider the strain tensor

$$E=\frac{1}{2}\left(H+H^{T}\right)\;\mathrm{or}\;E_{iK}=\frac{1}{2}\left(H_{iK}+H_{Ki}\right),$$

with $H_{iK}=\partial_{X_{K}}u_{i}$. Compute the time derivative,

$${\dot{E}}_{iK}=\frac{1}{2}\left({\dot{H}}_{iK}+{\dot{H}}_{Ki}\right)=\frac{1}{2}\left(\partial_{X_{K}}{\dot{u}}_{i}+\partial_{X_{i}}{\dot{u}}_{K}\right)=\frac{1}{2}\left(\partial_{x_{j}}{\dot{u}}_{i}F_{jK}+\partial_{x_{j}}{\dot{u}}_{K}F_{ji}\right).$$

Since F=1+H, then we find

$$\dot{E}=D+\mathrm{sym}\left(LH\right)$$

whence $\dot{E}≃D$ with an error sym(LH). Consequently, the assumption |H|≪1 justifies the approximation of the stress power as

$$T\cdot D≃T\cdot \dot{E}.$$

For technical convenience, the occurrence of the referential gradient ∇R in E, ∇RE, ∇R∇RE suggests that we consider the CD inequality (8) in a suitable referential form. Let $q_{R},k_{R}$ denote the referential heat flux and referential extra-entropy flux,

$$q_{R}=JqF^{-T},\;k_{R}=JkF^{-T}.$$

Observe that [4]

$$\nabla \;R\;f=\nabla f\;F,\;J\nabla \cdot k=\nabla \;R\;\cdot k_{R},\;Jq\cdot \nabla \theta =q_{R}\cdot \nabla \;R\;\theta .$$

Hence multiplying by *J* the CD inequality we find

(11) $$-\rho_{R}\left(\dot{\psi }+\eta \dot{\theta }\right)+JT\cdot \dot{E}+\theta \nabla \;R\;\cdot k_{R}-\frac{1}{\theta }q_{R}\cdot \nabla \;R\;\theta =\rho_{R}\theta \gamma .$$

Let

$$\Gamma =(\theta ,E,\nabla \;R\;E,\nabla \;R\;\nabla \;R\;E,\nabla \;R\;\theta ,\dot{E},\nabla \;R\;\dot{E})$$

be the set of variables while

$$\psi ,\eta ,T,k_{R},q_{R},\gamma$$

are given by (constitutive) functions of Γ. While $\eta ,T,q_{R},\gamma$ are assumed to be continuous the functions $\psi ,k_{R}$ are assumed to be continuously differentiable.

The spatial mass density ρ may be determined through $\rho_{R}$ by observing that

J=detF=det(1+H)≃1+trH=1+trE.

Consistent with the assumption |H|≪1 we may apply the approximation

$$\rho =\rho_{R}/J≃\rho_{R}\left(1-\mathrm{tr}\;E\right).$$

Compute $\dot{\psi }$ and substitute in (11) to obtain

(12) $$\begin{array}{c} -\rho_{R}\left(\partial_{\theta }\psi +\eta \right)\dot{\theta }-\rho_{R}\left(\partial_{E}\psi \cdot \dot{E}+\partial_{\nabla \;R\;E}\psi \cdot \nabla \;R\;\dot{E}+\partial_{\nabla \;R\;\nabla \;R\;E}\psi \cdot \nabla \;R\;\nabla \;R\;\dot{E}\right)+JT\cdot \dot{E}\\ -\rho_{R}\partial_{\nabla \;R\;\theta }\psi \cdot \nabla \;R\;\dot{\theta }-\rho_{R}\partial_{\dot{E}}\psi \cdot \ddot{E}-\rho_{R}\partial_{\nabla \;R\;\dot{E}}\psi \cdot \nabla \;R\;\ddot{E}+\theta \nabla \;R\;\cdot k_{R}-\frac{1}{\theta }q_{R}\nabla \;R\;\theta =\rho_{R}\theta \gamma . \end{array}$$

The linearity and arbitrariness of $\nabla \;R\;\ddot{E},\ddot{E},\nabla \;R\;\dot{\theta },\dot{\theta }$ imply that

(13) $$\partial_{\nabla \;R\;\dot{E}}\psi =0,\;\partial_{\dot{E}}\psi =0,\;\partial_{\nabla \;R\;\theta }\psi =0,\;\eta =-\partial_{\theta }\psi .$$

Divide by θ the remaining equation to have

(14) $$\begin{array}{c} -\frac{\rho_{R}}{\theta }\left(\partial_{E}\psi \cdot \dot{E}+\partial_{\nabla \;R\;E}\psi \cdot \nabla \;R\;\dot{E}+\partial_{\nabla \;R\;\nabla \;R\;E}\psi \cdot \nabla \;R\;\nabla \;R\;\dot{E}\right)\\ +\frac{J}{\theta }T\cdot \dot{E}+\nabla \;R\;\cdot k_{R}-\frac{1}{\theta^{2}}q_{R}\nabla \;R\;\theta =\rho_{R}\gamma . \end{array}$$

Using the identities

$$\begin{array}{c} \frac{\rho_{R}}{\theta }\partial_{\nabla \;R\;E}\psi \cdot \nabla \;R\;\dot{E}=\nabla \;R\;\cdot \left(\frac{\rho_{R}}{\theta }\partial_{\nabla \;R\;E}\psi \dot{E}\right)-\left[\nabla \;R\;\cdot \left(\frac{\rho_{R}}{\theta }\partial_{\nabla \;R\;E}\psi \right)\right]\cdot \dot{E},\\ \frac{\rho_{R}}{\theta }\partial_{\nabla \;R\;\nabla \;R\;E}\psi \cdot \nabla \;R\;\nabla \;R\;\dot{E}=\nabla \;R\;\cdot \left(\frac{\rho_{R}}{\theta }\partial_{\nabla \;R\;\nabla \;R\;E}\psi \nabla \;R\;\dot{E}\right)\\ -\nabla \;R\;\cdot \{\left[\nabla \;R\;\left(\nabla \;R\;\cdot \left(\frac{\rho_{R}}{\theta }\partial_{\nabla \;R\;\nabla \;R\;E}\psi \right)\right]\dot{E}\right\}+\left\{\nabla \;R\;\cdot \left[\nabla \;R\;\cdot \left(\frac{\rho_{R}}{\theta }\partial_{\nabla \;R\;\nabla \;R\;E}\psi \right)\right]\right\}\cdot \dot{E}, \end{array}$$

we can write Equation (14) in the form

(15) $$\left(-\frac{\rho_{R}}{\theta }\delta_{E}^{2}\psi +\frac{J}{\theta }T\right)\cdot \dot{E}+\nabla \;R\;\cdot \left(k_{R}-k_{E}\right)-\frac{1}{\theta^{2}}q_{R}\cdot \nabla \;R\;\theta =\rho_{R}\gamma ,$$

where $\delta_{E}^{2}$ is a second-order variational derivative defined by

(16) $$\delta_{E}^{2}\psi =\partial_{E}\psi -\frac{\theta }{\rho_{R}}\nabla \;R\;\cdot \left(\frac{\rho_{R}}{\theta }\partial_{\nabla \;R\;E}\psi \right)+\frac{\theta }{\rho_{R}}\nabla \;R\;\cdot \left[\nabla \;R\;\cdot \left(\frac{\rho_{R}}{\theta }\partial_{\nabla \;R\;\nabla \;R\;E}\psi \right)\right],$$

while

$$k_{E}=\frac{\rho_{R}}{\theta }\left[\left(\partial_{\nabla \;R\;E}\psi \right)\dot{E}+\left(\partial_{\nabla \;R\;\nabla \;R\;E}\psi \right)\nabla \;R\;\dot{E}\right]+\left[\nabla \;R\;\cdot \left(\frac{\rho_{R}}{\theta }\partial_{\nabla \;R\;\nabla \;R\;E}\psi \right)\right]\dot{E}.$$

By (13) it follows that $\delta_{E}^{2}$ is independent of $\dot{E},\nabla \;R\;\dot{E}$, and ∇Rθ. Hence the CD inequality (15) holds if

(17) $$T=\rho \delta_{E}^{2}\psi ,\;k_{R}=k_{E}$$

and

$$q_{R}\cdot \nabla \;R\;\theta =\rho_{R}\theta^{2}\gamma .$$

The result (17) shows that, though the CD inequality places severe restrictions on the constitutive functions, the single Cauchy stress tensor T is allowed to be a nonlinear function of the strain E and the strain gradients ∇RE,∇R∇RE. Furthermore, the present procedure gives evidence that the dependence on higher-order strain gradients would be consistent with thermodynamics. Technically, the stress T turns out to be a function of E,∇RE,∇R∇RE thanks to a non-zero extra-entropy $k_{R}$ and an appropriate free energy function ψ(θ,E,∇RE,∇R∇RE).

Yet a remark is in order. The present procedure involves the strain E and the gradients ∇RE,∇R∇RE thus allowing for the commutative properties

$${\left(\nabla \;R\;E\right)}^{˙}=\nabla \;R\;\dot{E},\;{\left(\nabla \;R\;\nabla \;R\;E\right)}^{˙}=\nabla \;R\;\nabla \;R\;\dot{E}.$$

By virtue of (17) the extra entropy flux $k_{R}$ is linear in $\dot{E}$ and $\nabla \;R\;\dot{E}$ and these terms are non-objective (see, e.g., [4] Section 1.9). Now, under the assumption |H|≪1, as a starting point we have used the approximation

$$T\cdot D≃T\cdot \dot{E},$$

on the stress power. As a conclusion, we can say that $k_{R}$ becomes objective if $\dot{E}$ is replaced with D.

As shown in this section, and likewise in the next one, the thermodynamic consistency of strain-gradient models is obtained by means of an extra-entropy flux that depends on $\dot{E}$ and $\nabla \;R\;\dot{E}$. This is so because the dependence of the free energy on the strain gradients leads to the time derivative of strain gradients, and then integration by parts produces the variational derivative of the stress tensor and an extra-entropy flux proportional to $\dot{E}$ and $\nabla \;R\;\dot{E}$. From the mechanical side, the power in the CD inequality is proportional to D or $\dot{E}$, and then the validity of the CD inequality holds if the extra entropy flux comprises suitable terms in $\dot{E}$ or $\nabla \;R\;\dot{E}$.

### 4.2. Piola Stress Through Green–Lagrange Strain Gradients

For definiteness we look for fourth-grade elastic solids in that the strain gradients involved are allowed up to fourth-order. Hence we let

$$\theta ,\nabla \;R\;\theta ,\nabla \;R\;\nabla \;R\;\theta ,E,\dot{E},\nabla \;R\;E,\nabla \;R\;\dot{E},\nabla \;R\;\nabla \;R\;E,\nabla \;R\;\nabla \;R\;\nabla \;R\;E,\nabla \;R\;\nabla \;R\;\nabla \;R\;\nabla \;R\;E,$$

be the variables and

$$\psi ,\eta ,T_{RR},q_{R},k_{R},\gamma$$

be the constitutive functions. For formal simplicity we let the free energy depend on strain gradients up to second order (and on the temperature gradient at the first order), namely

(18) ψ=ψ(θ,E,∇Rθ,∇RE,∇R∇RE).

Assume the function ψ is continuously differentiable while $\eta ,T_{RR},q_{R},k_{R},\gamma$ are continuous. Using the Coleman–Noll procedure [1], we now establish the thermodynamic requirements for fourth-grade materials with a free energy in the form of (18). Upon computation of $\dot{\psi }$ and substitution in the CD inequality we have

(19) $$\begin{array}{c} -\rho_{R}\left(\partial_{\theta }\psi +\eta \right)\dot{\theta }-\rho_{R}\partial_{\nabla \;R\;\theta }\psi \cdot \nabla \;R\;\dot{\theta }-\rho_{R}\partial_{E}\psi \cdot \dot{E}-\rho_{R}\partial_{\nabla \;R\;E}\psi \cdot \nabla \;R\;\dot{E}\\ -\rho_{R}\partial_{\nabla \;R\;\nabla \;R\;E}\psi \cdot \nabla \;R\;\nabla \;R\;\dot{E}+T_{RR}\cdot \dot{E}-\frac{1}{\theta }q_{R}\cdot \nabla \;R\;\theta +\theta \nabla \;R\;\cdot k_{R}=\rho_{R}\theta \gamma \geq 0. \end{array}$$

The linearity and arbitrarinesss of $\dot{\theta },\nabla \;R\;\dot{\theta }$ implies

$$\eta =-\partial_{\theta }\psi ,\;\partial_{\nabla \;R\;\theta }\psi =0,$$

whereas the values of $\nabla \;R\;\dot{E},\nabla \;R\;\nabla \;R\;\dot{E}$ cannot be regarded as (arbitrary and) independent of the other terms in the CD inequality, particularly in the expression of $\nabla \;R\;\cdot k_{R}$. Accordingly we divide Equation (19) by θ to have

(20) $$\begin{array}{c} \frac{1}{\theta }\left(T_{RR}-\rho_{R}\partial_{E}\psi \right)\cdot \dot{E}-\frac{\rho_{R}}{\theta }\partial_{\nabla \;R\;E}\psi \cdot \nabla \;R\;\dot{E}-\frac{\rho_{R}}{\theta }\partial_{\nabla \;R\;\nabla \;R\;E}\psi \cdot \nabla \;R\;\nabla \;R\;\dot{E}\\ -\frac{1}{\theta^{2}}q_{R}\cdot \nabla \;R\;\theta +\nabla \;R\;\cdot k_{R}=\rho_{R}\gamma \geq 0. \end{array}$$

In order to simplify the mathematical formalism we consider

(21) $$\Psi_{R}=\rho_{R}\psi /\theta .$$

The function $\Psi_{R}$ is the opposite of the Massieu potential [28]; borrowing from the terminology in [29], we can say that $\Psi_{R}$ is the Helmoltz free negentropy. Using (21) we consider the identities

$$\begin{array}{c} -\frac{\rho_{R}}{\theta }\partial_{\nabla \;R\;E}\psi \cdot \nabla \;R\;\dot{E}=-\partial_{\nabla \;R\;E}\Psi_{R}\cdot \nabla \;R\;\dot{E}=-\nabla \;R\;\cdot \left(\partial_{\nabla \;R\;E}\Psi_{R}\dot{E}\right)+\left[\nabla \;R\;\cdot \left(\partial_{\nabla \;R\;E}\Psi_{R}\right)\right]\cdot \dot{E},\\ -\frac{\rho_{R}}{\theta }\partial_{\nabla \;R\;\nabla \;R\;E}\psi \cdot \nabla \;R\;\nabla \;R\;\dot{E}=-\nabla \;R\;\cdot \left(\partial_{\nabla \;R\;\nabla \;R\;E}\Psi_{R}\nabla \;R\;\dot{E}\right)+\left[\nabla \;R\;\cdot \left(\partial_{\nabla \;R\;\nabla \;R\;E}\Psi_{R}\right)\right]\cdot \nabla \;R\;\dot{E}\\ =-\nabla \;R\;\cdot \left\{\partial_{\nabla \;R\;\nabla \;R\;E}\Psi_{R}\nabla \;R\;\dot{E}-\left[\nabla \;R\;\cdot \left(\partial_{\nabla \;R\;\nabla \;R\;E}\Psi_{R}\right)\right]\dot{E}\right\}-\left\{\nabla \;R\;\cdot \left[\nabla \;R\;\cdot \left(\partial_{\nabla \;R\;\nabla \;R\;E}\Psi_{R}\right)\right]\right\}\cdot \dot{E}, \end{array}$$

and we introduce the second-order variational derivative $\delta_{E}^{2}$ defined by

(22) $$\delta_{E}^{2}\Psi_{R}=\partial_{E}\Psi_{R}-\nabla \;R\;\cdot \left(\partial_{\nabla \;R\;E}\Psi_{R}\right)+\nabla \;R\;\cdot \left[\nabla \;R\;\cdot \left(\partial_{\nabla \;R\;\nabla \;R\;E}\Psi_{R}\right)\right].$$

Note that here $\delta_{E}^{2}$ can be exactly viewed as the standard variational derivative of order 2 with respect to E (see, e.g., [30] Chapter 4) in connection with the functional

$$I=\int_{\Omega }\Psi_{R}dv=\int_{\Omega }\frac{\rho_{R}}{\theta }\psi dv.$$

Accordingly, (20) can be rewritten as

(23) $$\frac{1}{\theta }{\hat{T}}_{RR}\cdot \dot{E}-\frac{1}{\theta^{2}}q_{R}\cdot \nabla \;R\;\theta +\nabla \;R\;\cdot \left(k_{R}-k_{E}\right)=\rho_{R}\gamma \geq 0,$$

where

(24) $${\hat{T}}_{RR}=T_{RR}-\theta \delta_{E}^{2}\Psi_{R},$$

$$k_{E}=\left[\partial_{\nabla \;R\;E}\Psi_{R}-\nabla \;R\;\cdot \left(\partial_{\nabla \;R\;\nabla \;R\;E}\Psi_{R}\right)\right]\dot{E}+\left(\partial_{\nabla \;R\;\nabla \;R\;E}\Psi_{R}\right)\nabla \;R\;\dot{E}.$$

Sufficient conditions for the validity of (23) with γ≥0 determine particular thermodynamically consistent models. In this sense a simple case arises by letting

$${\hat{T}}_{RR}=A\dot{E}-\theta \nabla_{R}\cdot \left(B\nabla_{R}\dot{E}\right),\;q_{R}=-K\nabla \;R\;\theta ,\;k_{R}=k_{E}-\left(B\nabla \;R\;\dot{E}\right)\dot{E},$$

where A,B and K are semi-positive definite tensors of fourth, sixth and second order, respectively. The corresponding expression of the entropy production is

$$\rho_{R}\gamma =\frac{1}{\theta }A\dot{E}\cdot \dot{E}+B\nabla \;R\;\dot{E}\cdot \nabla \;R\;\dot{E}+\frac{1}{\theta^{2}}K\nabla \;R\;\theta \cdot \nabla \;R\;\theta .$$

Restrict attention to a non-dissipative Piola stress $T_{RR}$. Hence we assume A=B=0 so that ${\hat{T}}_{RR}=0$, $k_{R}=k_{E}$, and from (24) we obtain

(25) $$T_{RR}=\theta \delta_{E}^{2}\Psi_{R}.$$

In suffix notation, this constitutive equation takes the form

$${\left(T_{RR}\right)}_{HK}=\rho_{R}\partial_{E_{HK}}\psi -\theta \partial_{X_{P}}\left(\frac{\rho_{R}}{\theta }\partial_{E_{HK,P}}\psi \right)+\theta \partial_{X_{P}}\partial_{X_{Q}}\left(\frac{\rho_{R}}{\theta }\partial_{E_{HK,PQ}}\psi \right)$$

By definition, the Cauchy stress T is then found to be

$$T=J^{-1}FT_{RR}F^{T}=J^{-1}\theta F\left(\delta_{E}\Psi_{R}\right)F^{T};$$

in components

$$T_{ij}=\rho F_{iH}\left[\partial_{E_{HK}}\psi -\frac{\theta }{\rho_{R}}\partial_{X_{P}}\left(\frac{\rho_{R}}{\theta }\partial_{E_{HK,P}}\psi \right)+\frac{\theta }{\rho_{R}}\partial_{X_{P}}\partial_{X_{Q}}\left(\frac{\rho_{R}}{\theta }\partial_{E_{HK,PQ}}\psi \right)\right]F_{Kj}^{T}.$$

A sufficient condition for the validity of (23) with γ≥0 arises by letting

$$q_{R}=-K\nabla \;R\;\theta ,\;k_{R}=k_{E},\;\rho_{R}\gamma =\frac{1}{\theta^{2}}K\nabla \;R\;\theta \cdot \nabla \;R\;\theta .$$

where K is a semi-positive definite tensor of second order. It is therefore evident that dissipation γ is only due to the heat flux, whereas the extra entropy flux vector $k_{R}$ is induced by the dependence of ψ on the strain gradients and vanishes if the strain is time-independent. In general, however, the strain rate $\dot{E}$ is not constrained in any way.

### 4.3. Some Examples

If θ and $\rho_{R}$ are spatially uniform then the $\theta /\rho_{R}$ factor can commute with the spatial operator $\partial_{X}$ so that

$$T_{ij}=\rho F_{iH}\left[\partial_{E_{HK}}\psi -\partial_{X_{P}}\partial_{\partial_{X_{P}}E_{HK}}\psi +\partial_{X_{P}}\partial_{X_{Q}}\left(\partial_{\partial_{X_{P}}\partial_{X_{Q}}E_{HK}}\psi \right)\right]F_{Kj}^{T}.$$

As an example, if

(26) $$\psi =\psi_{0}\left(\theta \right)+\frac{1}{2}{a|E|}^{2}+\frac{1}{2}{b|\nabla \;R\;E|}^{2}+\frac{1}{2}c{\left|\nabla \;R\;\nabla \;R\;E\right|}^{2},$$

where a,b, and *c* are spatially uniform parameters, then

(27) $$T_{ij}=\rho F_{iH}\left(aE_{HK}-b\Delta_{R}E_{HK}+c\Delta_{R}\Delta_{R}E_{HK}\right)F_{Kj}^{T}.$$

The symmetry of $T_{RR}$ and T is apparent from the thermodynamic requirement (25) and, necessarily, in the selected examples.

If a,b, and *c* are independent of θ in the free energy (26) then

$$\eta =-\psi_{0}^{\prime }\left(\theta \right).$$

Consequently, the internal energy ε=ψ+θη is given by

$$\varepsilon =\psi_{0}-\theta \psi_{0}^{\prime }\left(\theta \right)+\frac{1}{2}{a|E|}^{2}+\frac{1}{2}{b|\nabla \;R\;E|}^{2}+\frac{1}{2}c{\left|\nabla \;R\;\nabla \;R\;E\right|}^{2}.$$

Otherwise, a free energy of the form (26) with *a* independent of temperature but *b* and *c* proportional to θ, say $b=b_{0}\theta$ and $c=c_{0}\theta$, gives an entropy function of the form

$$\eta =-\psi_{0}^{\prime }\left(\theta \right)+\frac{1}{2}b_{0}{\left|\nabla \;R\;E\right|}^{2}+\frac{1}{2}c_{0}{\left|\nabla \;R\;\nabla \;R\;E\right|}^{2}$$

and then the internal energy

$$\varepsilon =\psi_{0}-\theta \psi_{0}^{\prime }\left(\theta \right)+\frac{1}{2}a{\left|E\right|}^{2}$$

is independent of strain gradients.

A more involved example arises by letting the Helmholtz free energy $\rho_{R}\psi$ be defined as follows (compare with Equation (18) in [6])

(28) $$\rho_{R}\psi =\frac{1}{2}E\cdot CE+\frac{1}{2}\nabla \;R\;E\cdot G\nabla \;R\;E+\frac{1}{2}\nabla \;R\;\nabla \;R\;E\cdot H\nabla \;R\;\nabla \;R\;E+\frac{1}{2}E\cdot K\nabla \;R\;\nabla \;R\;E.$$

In components

$$\begin{array}{cc} \rho_{R}\psi & =\frac{1}{2}E_{ij}C_{ijhk}E_{hk}+\frac{1}{2}\left(\partial_{X_{i}}E_{jh}\right)G_{ijhkmn}\left(\partial_{X_{k}}E_{mn}\right)\\ & +\frac{1}{2}\left(\partial_{X_{i}}\partial_{X_{j}}E_{hk}\right)H_{ijhkmnpq}\left(\partial_{X_{k}}\partial_{X_{m}}E_{pq}\right)+\frac{1}{2}E_{ij}K_{ijhkmn}\left(\partial_{X_{h}}\partial_{X_{k}}E_{mn}\right), \end{array}$$

where

$$C_{ijhk}=C_{jikh}=C_{hkij},\;G_{ijhkmn}=G_{ihjknm}=G_{kmnijh},$$

$$H_{ijhkmnpq}=H_{jikhnmqp}=H_{mnpqijhk},\;K_{ijhkmn}=K_{jikhnm}.$$

The corresponding Helmholtz free negentropy reads

$$\Psi_{R}=\frac{1}{2}E\cdot \tilde{C}E+\frac{1}{2}\nabla \;R\;E\cdot \tilde{G}\nabla \;R\;E+\frac{1}{2}\nabla \;R\;\nabla \;R\;E\cdot \tilde{H}\nabla \;R\;\nabla \;R\;E+\frac{1}{2}E\cdot \tilde{K}\nabla \;R\;\nabla \;R\;E,$$

where a superposed tilde denotes division by θ. These assumptions imply

$$\begin{array}{cc} {\left(\partial_{E}\Psi_{R}\right)}_{ij} & ={\tilde{C}}_{ijhk}E_{hk}+\frac{1}{2}{\tilde{K}}_{ijhkmn}\left(\partial_{X_{h}}\partial_{X_{k}}E_{mn}\right),\;{\left(\partial_{\nabla \;R\;E}\Psi_{R}\right)}_{ijh}={\tilde{G}}_{ijhkmn}\left(\partial_{X_{k}}E_{mn}\right),\\ {\left(\partial_{\nabla \;R\;\nabla \;R\;E}\Psi_{R}\right)}_{ijhk} & ={\tilde{H}}_{ijhkmnpq}\left(\partial_{X_{k}}\partial_{X_{m}}E_{pq}\right)+\frac{1}{2}{\tilde{K}}_{ijhkmn}^{T}E_{mn}, \end{array}$$

where ${\tilde{K}}_{ijhkmn}^{T}={\tilde{K}}_{mnijhk}$. From the general form (28) we obtain

$$\begin{array}{cc} T_{1} & :=\partial_{E}\Psi_{R}=\tilde{C}E+\frac{1}{2}\tilde{K}\nabla \;R\;\nabla \;R\;E,\;T_{2}:=\partial_{\nabla \;R\;E}\Psi_{R}=\tilde{G}\nabla \;R\;E,\\ T_{3} & :=\partial_{\nabla \;R\;\nabla \;R\;E}\Psi_{R}=\tilde{H}\nabla \;R\;\nabla \;R\;E+\frac{1}{2}{\tilde{K}}^{T}E. \end{array}$$

Then, due to (22), the stress formula (25) gives (see Equation (12) in [6])

(29) $$\begin{array}{cc} \frac{1}{\theta }T_{RR} & =T_{1}-\nabla \;R\;\cdot T_{2}+\nabla \;R\;\cdot \left(\nabla \;R\;\cdot T_{3}\right)=\tilde{C}E+\frac{1}{2}\tilde{K}\nabla \;R\;\nabla \;R\;E\\ & -\nabla \;R\;\cdot \left(\tilde{G}\nabla \;R\;E\right)+\nabla \;R\;\cdot \left[\nabla \;R\;\cdot \left(\tilde{H}\nabla \;R\;\nabla \;R\;E+\frac{1}{2}{\tilde{K}}^{T}E\right)\right]. \end{array}$$

Though the Lagrangian scheme for the strain-gradient is quite unusual in the literature, by analogy with other schemes, one might say that CE represents the local linear form of the Piola stress, while $\theta T_{1}-CE$, $\theta T_{2}$ and $\theta T_{3}$ denote hyperstresses (of different order). Apart from the interpretation of the tensors $T_{1}$, $T_{2}$, and $T_{3}$, here we conclude that the effective stress, entering the equation of motion, involves the strain and even-order spatial derivatives of strain.

## 5. Variational Approaches to Higher-Order Strain Gradients

Higher-order strain gradient theories derive the equation of motion by applying Hamilton’s principle (see, e.g., Refs. [6,21,31] and references therein). Also in view of the difference with the previous Cauchy-like approaches, it is worth revisiting the whole procedure. Let K and W be the kinetic energy density and the strain energy density. Hence

L=K−W

is the Lagrangian density. By analogy with, e.g., [6,7,23], we assume

W=W(ϵ,∇∇u,∇∇∇u).

As in [6], we let

$$K=K(\dot{u},\nabla \dot{u},\nabla \nabla \dot{u}).$$

The unknown function is the displacement u(x,t), with $x\in \Omega ,t\in [t_{1},t_{2}]$. Consider the (action) functional

$$I=\int_{t_{1}}^{t_{2}}\int_{\Omega }Ldv\;dt+\int_{t_{1}}^{t_{2}}\int_{\Omega }f\cdot u\;dv\;dt+\int_{t_{1}}^{t_{2}}\int_{\partial \Omega }t\cdot u\;da\;dt,$$

where f is the external force per unit volume and t is the external traction. The unknown field u is assumed to satisfy Hamilton’s principle in that u+αh, with $h\left(t_{1}\right)=h\left(t_{2}\right)=0$, makes *I* stationary at α=0. Letting ${D=d/d\alpha |}_{\alpha =0}$ (see, e.g., [32] Section 4.8) then we assume Hamilton’s principle in the form

(30) DI(u+αh)=0.

To find the consequences of (30) we notice that as u→u+αh then

$$\dot{u}\to \dot{u}+\alpha \dot{h},\;\epsilon \to \epsilon +\alpha \lambda ,\;\lambda =\mathrm{sym}\nabla h.$$

Hence

$$\begin{array}{c} DI=\int_{t_{1}}^{t_{2}}\int_{\Omega }\{\partial_{\dot{u}}K\cdot \dot{h}-\partial_{\epsilon }W\cdot \lambda -\partial_{\nabla \nabla u}W\cdot \nabla \nabla h-\partial_{\nabla \nabla \nabla u}W\cdot \nabla \nabla \nabla h\\ +f\cdot h\}dv\;dt+\int_{t_{1}}^{t_{2}}\int_{\partial \Omega }t\cdot h\;da\;dt, \end{array}$$

Let

$$\tau_{ij}=\partial_{\epsilon_{ij}}W,\;T_{ijk}=\partial_{u_{i,jk}}W,\;S_{ijpk}=\partial_{u_{i,jpk}}W$$

with the obvious symmetries

$$\tau_{ij}=\tau_{ji},\;T_{ijk}=T_{ikj},\;S_{ijpk}=S_{ipjk}=S_{ijkp}=S_{ikpj}.$$

Using the identities

$$\partial_{\partial_{t}u}K\cdot \partial_{t}h=\partial_{t}\left(\partial_{\partial_{t}u}K\cdot h\right)-\left(\partial_{t}\partial_{\partial_{t}u}K\right)\cdot h,$$

$$\tau_{ij}\lambda_{ij}={\left(h_{i}\tau_{ij}\right)}_{,j}-\tau_{ij,j}h_{i},$$

$$T_{ijk}h_{i,jk}={\left(T_{ijk}h_{i,j}\right)}_{,k}-{\left(T_{ijk,k}h_{i}\right)}_{,j}+T_{ijk,jk}h_{i},$$

$$S_{ijpk}h_{i,jpk}={\left(S_{ijpk}h_{i,jp}\right)}_{,k}-{\left(S_{ijpk,k}h_{i,j}\right)}_{,p}+{\left(S_{ijpk,pk}h_{i}\right)}_{,j}-S_{ijpk,jpk}h_{i}$$

we can write the vanishing of DI in the form

(31) $$\begin{array}{c} 0=DI=\int_{\Omega }\left(\partial_{\partial_{t}u}K\cdot h\right){|}_{t_{1}}^{t_{2}}dv\\ +\int_{t_{1}}^{t_{2}}\int_{\Omega }\left[-\partial_{t}\partial_{\partial_{t}u_{i}}K+\tau_{ij,j}-T_{ijk,jk}+S_{ijpk,jpk}+f_{i}\right]h_{i}\;dv\;dt\\ +\int_{t_{1}}^{t_{2}}\int_{\partial \Omega }\left[\left(-\tau_{ij}+T_{ijk,k}-S_{ijpk,pk}\right)n_{j}+t_{i}\right]h_{i}\;da\;dt\\ +\int_{t_{1}}^{t_{2}}\int_{\partial \Omega }\left[\left(-T_{ijk}+S_{ijkp,p}\right)H_{i,j}-S_{ijpk}h_{i,jp}\right]n_{k}. \end{array}$$

By the arbitrariness of h we can take h,∇h and ∇∇h to be zero at the boundary ∂Ω and h=0 at $t_{1},t_{2}$. Hence it follows that DI=0 implies

$$\int_{t_{1}}^{t_{2}}\int_{\Omega }\left[-\partial_{t}\partial_{\partial_{t}u_{i}}K+\tau_{ij,j}-T_{ijk,jk}+S_{ijpk,jpk}+f_{i}\right]h_{i}\;dv\;dt=0.$$

The arbitrariness of h implies that

(32) $$-\partial_{t}\partial_{\partial_{t}u_{i}}K+\tau_{ij,j}-T_{ijk,jk}+S_{ijpk,jpk}+f_{i}=0.$$

If we let K be the usual kinetic energy,

$$K=\frac{1}{2}\rho_{0}{\left(\partial_{t}u\right)}^{2},$$

we find that (32) is the equation of motion,

(33) $$\rho_{0}\partial_{t}^{2}u_{i}=\tau_{ij,j}-T_{ijk,jk}+S_{ijpk,jpk}+f_{i}$$

with

(34) $$T_{ij}=\tau_{ij}-T_{ijk,k}+S_{ijpk,pk}$$

as the effective stress tensor. It is worth remarking that the structure (34) of the effective stress tensor follows as in Section 3 without assuming from the start that the power involves the hyper-stresses.

Some comments are in order about the variational formulation. As is standard in the literature (see, e.g., [31,33]) the unknown fields are described as functions of x and *t*, as in Eulerian descriptions, with Ω being viewed as a control region. This however is open to the objection that $\partial_{t}u$ is not the velocity. To overcome this problem we might formally follow the same procedure with Ω as a convecting region and accordingly having the time derivative as the material one. Irrespective of the view on the region Ω, the mass density $\rho_{0}$ is involved as a constant.

## 6. Comparing the CD Inequality and Hamilton’s Principle

Some results, as here in (16)–(17) and (34), might indicate the CD inequality and Hamilton’s principle as alternative procedures. Yet the two procedures are conceptually and formally deeply different. Conceptually, Hamilton’s principle originates from the analytical mechanics and leads to balance equations (see, e.g., [33] Section 7.7.3). Instead, the CD inequality presupposes the balance equations and selects admissible constitutive equations.

Formally, the classical variation of the action Lagrangian δI, with

$$I=\int_{t_{1}}^{t_{2}}\int_{\Omega }L\;dv\;dt,$$

here replaced with the derivative DI, involves the variations of the unknown fields. The generic term δL is then

$$\partial_{u}L\cdot h$$

or

$$\partial_{\nabla u}L\cdot \nabla h.$$

Instead the CD inequality involves the material time derivative $\dot{\psi }$ of the pertinent potential function ψ and

$$\dot{\psi }=\partial_{\nabla u}\psi \cdot {\left(\nabla h\right)}^{˙}$$

while

$${\left(\nabla h\right)}^{˙}\neq \nabla \dot{h}.$$

Indeed, for a dependence on a gradient $\nabla \partial_{t}u$ the variation $\nabla \partial_{t}h$ satisfies the identity $\nabla \partial_{t}h=\partial_{t}\nabla h$ whereas the time derivative of ψ(∇u) provides

$${\left(\nabla h\right)}^{˙}\neq \nabla \dot{h}.$$

Furthermore, within a variational formulation, integration by parts results directly in boundary terms. In the CD inequality, the analogous boundary terms occur through the extra-entropy flux k, and this involves the temperature θ and the mass density ρ. Only if θ and ρ are uniform then the boundary terms in the two procedures are formally the same.

## 7. Conclusions

Among the modelling of non-local properties of elastic materials, the schemes involving strain gradients exhibit interesting conceptual problems. Owing to the elastic character, a dependence of the stress on strain gradients has to be inherited from an appropriate free energy potential. However, this in turn requires that the stress power be in the form of (9) with a corresponding sum of terms in the time derivative of the strain gradient. In some approaches the consistency of such a stress power is justified through the statement of the virtual power, with the single stress terms being called hyper-stresses. Quite a similar scheme follows through a variational statement (Section 5). In both cases the stress T takes the form (34) through the hyper-stresses T,S. Yet both approaches are outside any thermodynamic analysis.

Instead, as is shown in Section 4, a thermodynamic scheme is allowed with a (body) stress power T·D, without any appeal to hyper-stresses, by letting the free energy, and hence the stress, be a function of gradients of the Green–Lagrange strain tensor. An Eulerian version follows as an approximation of the Lagrangian scheme. The stress tensor is eventually found to have the form of a variational derivative, though with additional terms if the temperature field is not uniform. Indeed, it is shown that the dynamic equation arising from the thermodynamic restrictions equals the equation produced by a variational approach for the Massieu potential.

The result of this paper is that even for non-simple continua, as with strain-gradient elastic solids, a thermodynamically consistent model can be established by a detailed analysis of the second law of thermodynamics while maintaining the classical form of the balance laws.

As to other approaches to strain-gradient models, we mention [18] where the stress power involves a hyper-stress S and the equation of motion is assumed with T−∇·S as the effective stress. The formal difference is the dependence on the curvature tensor $F^{-1}\nabla_{R}F$ instead of merely $\nabla_{R}F$. In [16], Liu’s procedure is applied with the assumption that the internal energy *u* has the form

$$u=e-\frac{1}{2}v^{2}-\frac{1}{2}\alpha_{1}{\left[\mathrm{tr}\;\dot{F}\right]}^{2}-\frac{1}{2}\alpha_{2}\mathrm{tr}\left(\dot{F}\dot{F}\right),$$

where *e* is the standard internal energy. Aside from dissipative terms, it follows that the stress T is subject to the constitutive equation

$$T_{ij}-\alpha_{1}T_{lk,lk}\delta_{ij}-\alpha_{2}T_{ki,jk}=\partial_{F_{ij}}\psi -{\left(\partial_{F_{ij,k}}\psi \right)}_{,k}.$$

The unusual assumption on the internal energy leads to an unusual constitutive equation for the stress T that deserves particular attention.

## Data Availability

No new data have been created. The study did not report any data.

## Abbreviations

The following abbreviations are used in this manuscript:

CD	Clausius–Duhem
